# Supplementary material for: Blinded, randomized trial of sonographer versus AI cardiac function assessment
Source: Nature. 2023 Apr 5;616(7957):520–4. doi: 10.1038/s41586-023-05947-3 (PMC10115627; doi:10.1038/s41586-023-05947-3)
Supplement: Supplementary file 1 — This file contains the technical methods, Supplementary Figs. 1–3 and Supplementary Table 1. [file 41586_2023_5947_MOESM1_ESM.docx]

SUPPLEMENTARY MATERIALS AND RESULTS

TECHNICAL METHODS Page 2-3
SUPPLEMENTARY FIGURE 1: User Interface Page 4
SUPPLEMENTARY FIGURE 2: Model Performance vs. Training Dataset Size Page 5
SUPPLEMENTARY TABLE 1: Training Dataset Demographics Page 6
SUPPLEMENTARY FIGURE 3: Performance of individual sonographers vs. AI Page 7

**TECHNICAL METHODS**

The model architecture and workflow was previously published as:

# **Video-based AI for beat-to-beat assessment of cardiac function** David Ouyang, Bryan He, Amirata Ghorbani, Neal Yuan, Joseph Ebinger, Curt P. Langlotz, Paul A. Heidenreich, Robert A. Harrington, David H. Liang, Euan A. Ashley, and James Y. Zou. *Nature*, March 25, 2020. <https://doi.org/10.1038/s41586-020-2145-8>

The associated code for the model as well as trained weights are available at:

<https://github.com/echonet/dynamic>

A dataset of 10,030 de-identified echocardiogram videos used to train a model at:

[https://echonet.github.io/dynamic](https://echonet.github.io/dynamic/)

This supplemental appendix is meant to give an overview of the model as well as highlight changes from original Nature paper and additions in implementation for the clinical trial.

Our deep learning pipeline is trained to evaluate left ventricular ejection fraction (LVEF) and annotate the left ventricle from apical-2-chamber (A2C) and apical-4-chamber (A4C) echocardiogram videos originally stored as DICOM files. The LVEF is provided as a numerical output between 0 and 100, and the left ventricle annotation is provided as a binary mask of pixels within the left ventricle. For evaluating LVEF, our pipeline uses a video-based 3D convolutional neural network, and for annotating the left ventricle, our pipeline uses a semantic segmentation model. Our prior paper describes the workflow for training the models and the hyperparameter sweep used to select the model architecture, video frame rate, and clip length.

For the purposes of our clinical trial, four main technical additions were made for our AI model implementation. First, an echocardiogram view classifier model was trained to select A2C and A4C videos from full echocardiogram studies. As an end-to-end pipeline, the initial input is the full study of echocardiogram videos as DICOM files such that the model selects the optimal A2C and A4C view video. While clinical guidelines recommend using biplane LVEF measurements, sonographers varied in whether they annotated an A2C video. To maintain blinding, in the AI arm, the model output presented to the cardiologist was only the results from the A4C video if the sonographer only labeled the A4C video, and the biplane result was presented only if the sonographer labeled both views.

Second, while the originally published paper allowed for frame by frame annotation and beat by beat assessment of LVEF, this is not the standard clinical practice of sonographers, who primarily annotate one beat in each video. To maintain blinding, in the AI arm, the model output presented to the cardiologist was the most representative heartbeat. The beat with a calculated LVEF closest to the overall model estimate was selected as this representative heartbeat. Similarly, our model annotated the left ventricle with more detail than than human sonographers. To maintain blinding in the trial, the annotation points were subsampled to present a similar number of points as human sonographers.

Third, our model was fully embedded in the clinical reporting system software (Siemens Syngo VA40D) so there was no visual difference in user interface or method to adjust left ventricular ejection fraction (Supplemental Figure 1 and Supplemental Video). The binary mask of the semantic segmentation model was converted to a polygon representing the left ventricle in the backend SQL server of the reporting software. The reporting software’s internal structure and algorithms were used to specify the long axis of the ventricle and calculate ventricular volumes by method of disks. This way, any adjustments of either sonographer or AI annotations would result in the same workflow and calculations to update left ventricular ejection fraction.

Fourth, consistent with other works in machine learning, we found that model performance improvement scales with input training dataset size. As such, we collected a dataset consisting of 147,378 A2C and A4C echocardiogram videos stored as DICOM files from Stanford Healthcare to train and evaluate our model. Importantly, our model was never trained on any echocardiogram videos from Cedars-Sinai Medical Center and, as such, the clinical trial was done on an external test site with respect to data source. We split our dataset chronologically by study into training, validation, and test sets, and found no clear asymptote in the range of training set sizes available (Supplemental Figure 2). The demographics of the training set are described in Supplemental Table 1, and the demographics of the clinical trial are listed in the main manuscript (Table 1).

We finally provide details for processing the DICOM files from the Stanford Healthcare dataset and studies from Cedars-Sinai Medical Center used in the clinical trial, along with the process for training the deep learning models on the Stanford Healthcare dataset. The video data in the DICOM files is first cropped to a square window including the ultrasound window and then scaled to a resolution of 112 pixels x 112 pixels. Each frame of the video is represented as a 112 x 112 x 3 array of 8-bit unsigned integers. The models for evaluating LVEF and annotating the left ventricle are both initialized with pretrained weights trained on non-medical datasets. Both models are then trained on the Stanford Healthcare dataset. The model to evaluate LVEF is trained for 20 epochs with a learning rate of 1e-4 and a weight decay of 1e-4, with the learning rate decreased by a factor of 10 after 10 epochs, the model to annotate the left ventricle is trained for 10 epochs with a learning rate of 1e-6 and a weight decay of 0.


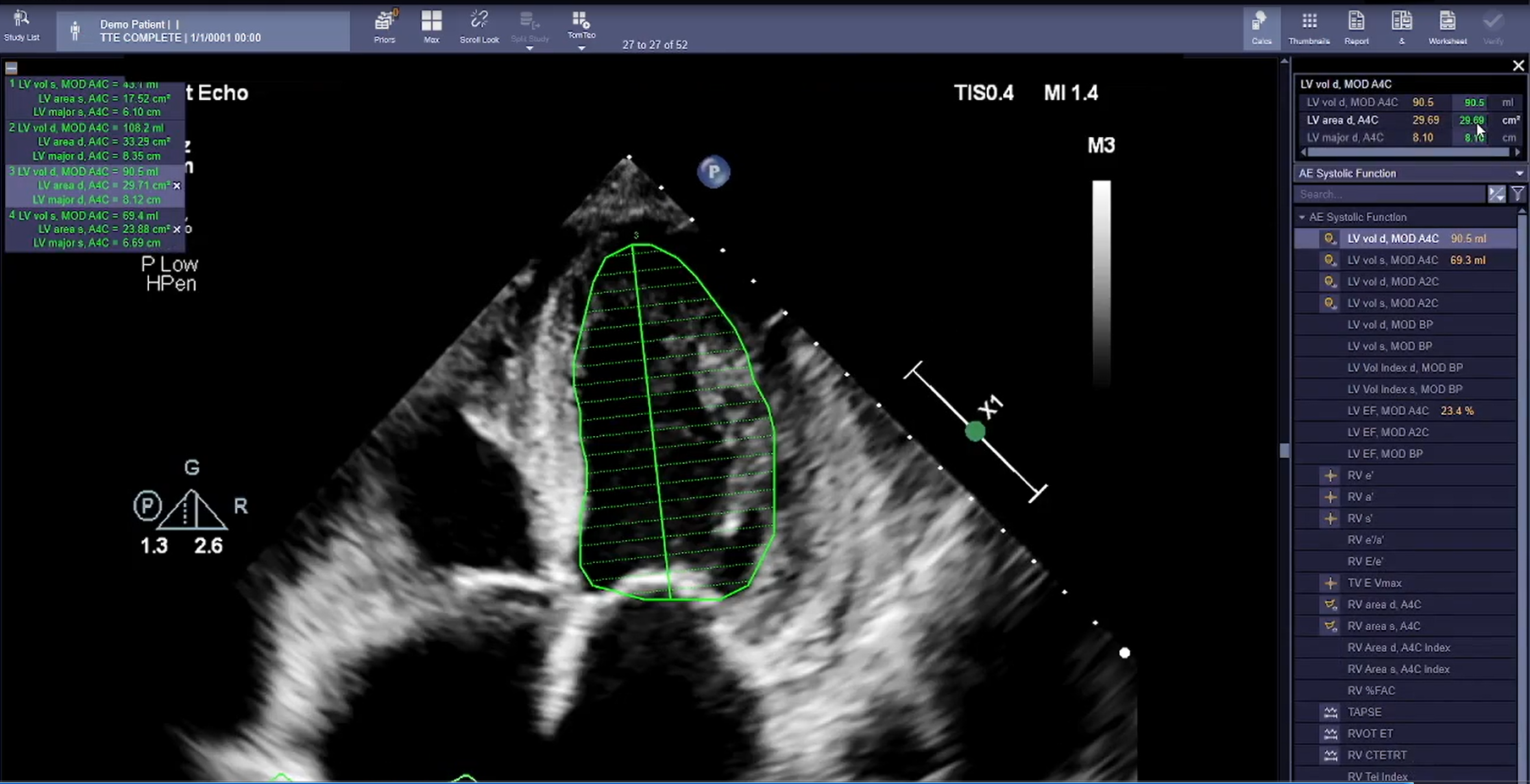


Supplemental Figure 1: User Interface for Cardiologist Assessment. Annotation of the left ventricle dynamically changes LVEF. Full transthoracic echocardiogram study presented to the cardiologist, with annotations on select A2C and A4C videos to optimize blinding compared to sonographers. In blinded trial, only one annotation (either by AI or sonographer) was presented per study.


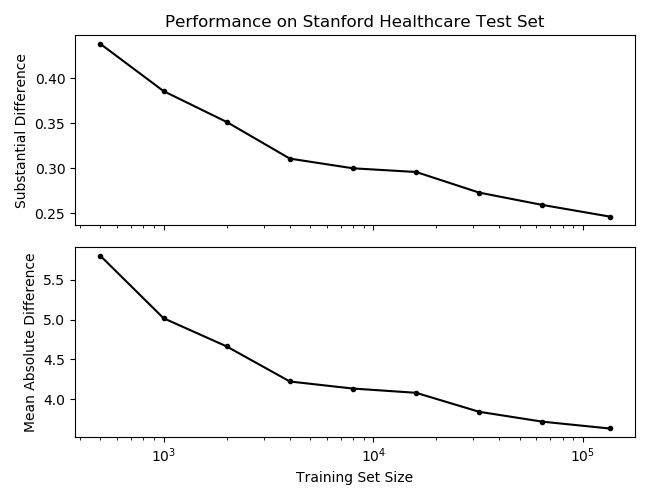


Supplemental Figure 2: Model Performance on Stanford Healthcare test set with varying training set size.

Supplemental Table 1: Stanford Training Data Characteristics

|  | Total | Train | Val | Test |
| --- | --- | --- | --- | --- |
| Variable | (n = 144184) | (n = 133177) | (n = 5102) | (n = 5105) |
| Age - yr * | 59.8 ± 17.7 | 59.6 ± 17.7 | 61.7 ± 17.4 | 61.5 ± 17.9 |
| Sex - no. (%) |  |  |  |  |
| Male | 67286 (46.7%) | 62552 (46.7%) | 2361 (46.3%) | 2373 (46.5%) |
| Female | 52162 (36.2%) | 48079 (35.9%) | 2029 (39.8%) | 2054 (40.2%) |
| Missing | 24736 (17.2%) | 23346 (17.4%) | 767 (14.0%) | 678 (13.3%) |
| Race - no. (%) |  |  |  |  |
| White | 64417 (44.7%) | 59892 (44.7%) | 2230 (43.7%) | 2295 (44.3%) |
| Black | 4900 (3.4%) | 4573 (3.4%) | 166 (3.3%) | 161 (3.2%) |
| Asian | 18777 (13.0%) | 17239 (12.9%) | 782 (15.4%) | 756 (14.8%) |
| Other | 22668 (15.7%) | 20884 (15.6%) | 908 (17.8%) | 876 (17.2%) |
| Pacific Islander | 1822 (1.3%) | 1680 (1.2%) | 58 (1.1%) | 84 (1.6%) |
| American Indian | 336 (0.2%) | 302 (0.2%) | 21 (0.4%) | 13 (0.3%) |
| Unknown | 31264 (21.7%) | 29407 (21.9%) | 937 (18.4%) | 920 (18.0%) |
| Ethnicity - no. (%) |  |  |  |  |
| Hispanic | 15703 (10.9%) | 14469 (10.8%) | 615 (12.1%) | 619 (12.1%) |
| Non-Hispanic | 97342 (67.5%) | 90180 (67.3%) | 3586 (70.2%) | 3586 (70.2%) |
| Unknown | 31139 (21.6%) | 29328 (21.9%) | 911 (17.9%) | 900 (17.6%) |
| Body Mass Index* | 26.6 ± 6.0 | 26.6 ± 6.0 | 26.9 ± 6.1 | 26.8 ± 6.2 |
| Prior Clinical EF | 55.1 ± 12.8 | 54.9 ± 12.9 | 56.6 ± 11.9 | 56.6 ± 11.6 |

*Age missing in 6120 videos, BMI missing in 28,120 videos


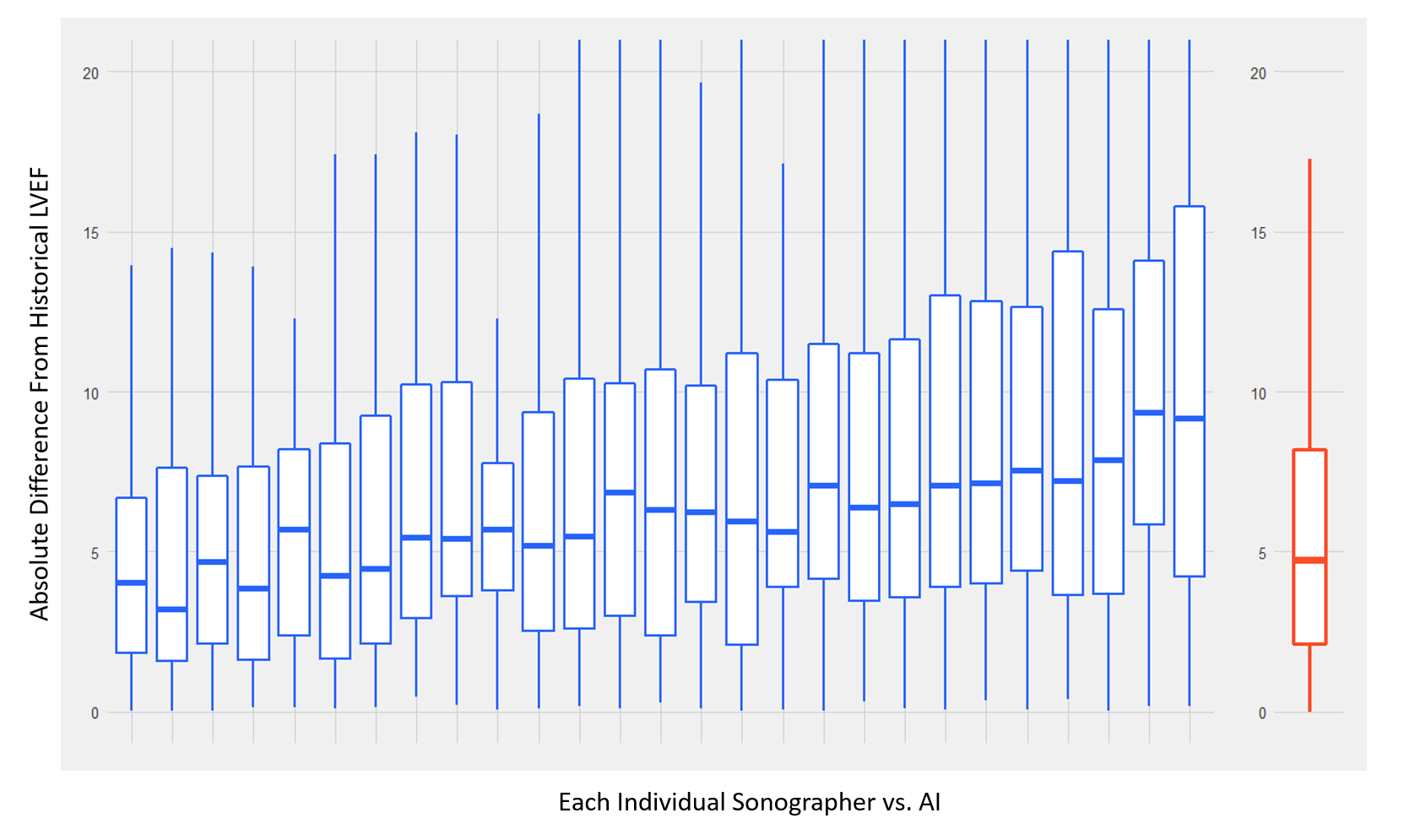
 Supplemental Figure 3: Performance of each individual sonographer vs. AI initial assessment compared to historical assessment. Boxplot represents the median as a thick line, interquartile range (IQR) of 25% and 75% as upper and lower bounds of the box, and whiskers to minimum and maximum difference up to 1.5*IQR. Sample size for each sonographer ranges from n = 15 to n = 114 and sample size of AI is n = 1740.
