# Supplementary material for: Blinded, randomized trial of sonographer versus AI cardiac function assessment
Source: Nature. 2023 Apr 5;616(7957):520–4. doi: 10.1038/s41586-023-05947-3 (PMC10115627; doi:10.1038/s41586-023-05947-3)
Supplement: Supplementary file 2 — Reporting Summary [file 41586_2023_5947_MOESM2_ESM.pdf]

nature portfolio

Corresponding author(s): Ouyang et al

Last updated by author(s): Oct 25, 2022

Reporting Summary

Nature Portfolio wishes to improve the reproducibility of the work that we publish. This form provides structure for consistency and transparency in reporting. For further information on Nature Portfolio policies, see our Editorial Policies and the Editorial Policy Checklist.

Statistics

For all statistical analyses, confirm that the following items are present in the figure legend, table legend, main text, or Methods section.

n/a	Confirmed	
☐	☒	The exact sample size (*n*) for each experimental group/condition, given as a discrete number and unit of measurement
☐	☒	A statement on whether measurements were taken from distinct samples or whether the same sample was measured repeatedly
☐	☒	The statistical test(s) used AND whether they are one- or two-sided<br>*Only common tests should be described solely by name; describe more complex techniques in the Methods section.*
☐	☒	A description of all covariates tested
☐	☒	A description of any assumptions or corrections, such as tests of normality and adjustment for multiple comparisons
☐	☒	A full description of the statistical parameters including central tendency (e.g. means) or other basic estimates (e.g. regression coefficient) AND variation (e.g. standard deviation) or associated estimates of uncertainty (e.g. confidence intervals)
☐	☒	For null hypothesis testing, the test statistic (e.g. *F*, *t*, *r*) with confidence intervals, effect sizes, degrees of freedom and *P* value noted<br>*Give P values as exact values whenever suitable.*
☒	☐	For Bayesian analysis, information on the choice of priors and Markov chain Monte Carlo settings
☒	☐	For hierarchical and complex designs, identification of the appropriate level for tests and full reporting of outcomes
☒	☐	Estimates of effect sizes (e.g. Cohen's *d*, Pearson's *r*), indicating how they were calculated

Our web collection on statistics for biologists contains articles on many of the points above.

Software and code

Policy information about availability of computer code

Data collection	Data was collected from the echocardiography laboratories at Stanford Healthcare and Cedars-Sinai Medical Center. Medical imaging were de-identified from initial DICOM files prior to processing by deep learning algorithm. The deep learning algorithm, written in Python with OpenCV (4.5.1.48), Pytorch (1.8.0) and Torchvision (0.9.0). A full list of dependencies is at https://github.com/echonet/dynamic/blob/master/requirements.txt. Code is available at https://github.com/echonet/dynamic, was used to assess the echocardiogram videos.
Data analysis	Statistical analysis is detailed in the statistical analysis plan, to be made available at: https://github.com/echonet/blinded_rct.<br>Analyses were performed in R 4.1.0.

For manuscripts utilizing custom algorithms or software that are central to the research but not yet described in published literature, software must be made available to editors and reviewers. We strongly encourage code deposition in a community repository (e.g. GitHub). See the Nature Portfolio guidelines for submitting code & software for further information.

Data

Policy information about availability of data

All manuscripts must include a data availability statement. This statement should provide the following information, where applicable:

- Accession codes, unique identifiers, or web links for publicly available datasets
- A description of any restrictions on data availability
- For clinical datasets or third party data, please ensure that the statement adheres to our policy

The AI model was trained using echocardiogram videos from Stanford Healthcare following Stanford IRB protocol 43721 with waiver of individual consent. A set of de-identified Stanford Healthcare echocardiogram videos is publicly available at https://echonet.github.io/dynamic/. The clinical trial was performed at Cedars-Sinai Medical Center under IRB protocol STUDY1707, and the study protocol, statistical analysis plan, and de-identified trial results will be available at https://github.com/echonet/blinded_rct. Data requests for identifiable data will be evaluated by the authors to maintain compliance with IRB protocol and data privacy protections. Please email david.ouyang@cshs.org for requests for identifiable data.

Human research participants

Policy information about studies involving human research participants and Sex and Gender in Research.

Reporting on sex and gender	Stratified data analysis was reported by sex and the cohort demographics were described in Table 1
Population characteristics	Detailed cohort characteristics given in Table 1 and Supplementary Table 1
Recruitment	Consecutive echocardiograms from August 2019 were to train the model. Sonographers and cardiologists from the Cedars Sinai Medical Center echo lab were recruited for the study and performed evaluation between February 1, 2022 and July 5, 2022.
Ethics oversight	The AI model was trained using echocardiogram videos from Stanford Healthcare following Stanford IRB protocol 43721 with waiver of individual consent. The clinical trial was performed at Cedars-Sinai Medical Center under IRB protocol STUDY1707

Note that full information on the approval of the study protocol must also be provided in the manuscript.

Field-specific reporting

Please select the one below that is the best fit for your research. If you are not sure, read the appropriate sections before making your selection.

☒ Life sciences ☐ Behavioural & social sciences ☐ Ecological, evolutionary & environmental sciences

For a reference copy of the document with all sections, see nature.com/documents/nr-reporting-summary-flat.pdf

Life sciences study design

All studies must disclose on these points even when the disclosure is negative.

Sample size	Non-inferiority Design: 8% vs. 5%, alpha of 0.05 and power of 0.9<br>2834 studies needed, pre-planned to enroll 3500 studies as buffer against dropout
Data exclusions	A pre-specified run in period was performed where sonographers traced all echocardiogram studies and studies that sonographers could not trace were excluded from randomization. All studies randomized were revaluated by cardiologists.
Replication	95% Confidence interval of reported metrics were determined by boot strapping. Trial was only performed once without replication.
Randomization	Studies were individually randomized 1:1 to AI vs. sonographer as agent of initial interpretation.
Blinding	Cardiologists were blinded to agent of initial interpretation and asked to guess agent of initial interpretation (AI vs. sonographer) in blinded fashion.

Reporting for specific materials, systems and methods

We require information from authors about some types of materials, experimental systems and methods used in many studies. Here, indicate whether each material, system or method listed is relevant to your study. If you are not sure if a list item applies to your research, read the appropriate section before selecting a response.

Materials & experimental systems

n/a	Involved in the study
☒	☐ Antibodies
☒	☐ Eukaryotic cell lines
☒	☐ Palaeontology and archaeology
☒	☐ Animals and other organisms
☐	☒ Clinical data
☒	☐ Dual use research of concern

Methods

n/a	Involved in the study
☒	☐ ChIP-seq
☒	☐ Flow cytometry
☒	☐ MRI-based neuroimaging

Clinical data

Policy information about clinical studies

All manuscripts should comply with the ICMJE guidelines for publication of clinical research and a completed CONSORT checklist must be included with all submissions.

Clinical trial registration: https://clinicaltrials.gov/ct2/show/NCT05140642

Study protocol: Study protocol in supplementary materials

Data collection: Data collected from Cedars Sinai Medical Center between February 2, 2021 and July 5, 2022

Outcomes:

Primary Outcome: Frequency and degree of change from initial (AI vs. sonographer) assessment to final cardiologist assessment
Substantial change defined as more than 5% LVEF

Secondary Outcomes:
Cardiologist Prediction of Agent of Initial Assessment (Blinding Assessment)
Sonographer Time
Cardiologist Time
Change from Historical Cardiologist Assessment
